# Selective Hydrodeoxygenation of Lignin-Derived Phenols to Aromatics Catalyzed by Nb_2_O_5_-Supported Iridium

**DOI:** 10.1021/acsomega.2c04314

**Published:** 2022-08-23

**Authors:** Gabriel Jeantelot, Simen P. Følkner, Johanna I. S. Manegold, Morten G. Ingebrigtsen, Vidar R. Jensen, Erwan Le Roux

**Affiliations:** Department of Chemistry, University of Bergen, Allégaten 41, N-5007 Bergen, Norway

## Abstract

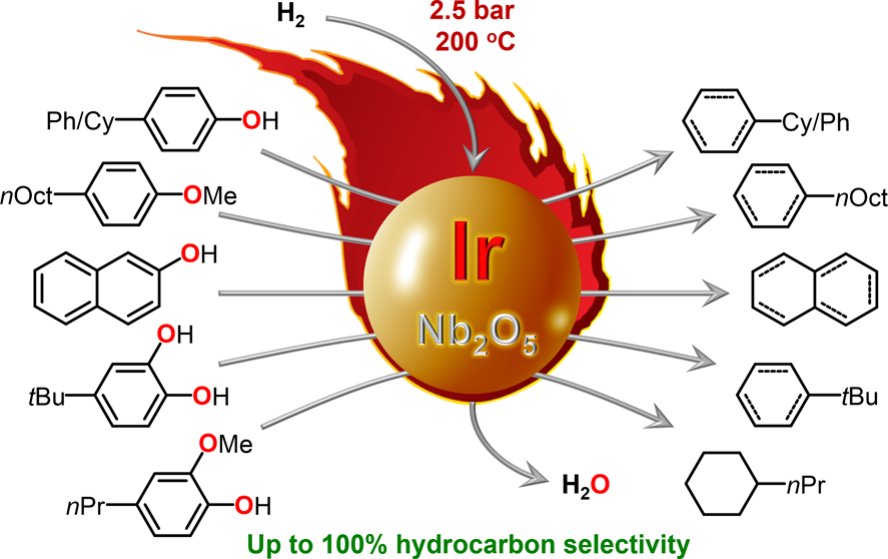

The dominating catalytic approach to aromatic hydrocarbons from renewables, deoxygenation of phenol-rich depolymerized lignin bio-oils, is hard to achieve: hydrodeoxygenation (HDO) of phenols typically leads to the loss of aromaticity and to non-negligible fractions of cyclohexanones and cyclohexanols. Here, we report a catalyst, niobia-supported iridium nanoparticles (Ir@Nb_2_O_5_), which combines full conversion in the HDO of lignin-derived phenols with appreciable and tunable selectivity for aromatics (25–95%) under mild conditions (200–300 °C, 2.5–10 bar of H_2_). A simple approach to the removal of Brønsted-acidic sites via Hünig’s base prevents coking and allows reaction conditions (*T* > 225 °C, 2.5 bar of H_2_), promoting high yields of aromatic hydrocarbons.

## 1 Introduction

Lignin is a phenolic polymer, which makes up a considerable fraction of wood and plant matter, and is a significant waste product of the pulp and paper industry.^1^ Its breakdown into discrete molecules therefore makes it a uniquely abundant source of renewable hydrocarbons, aromatic hydrocarbons, in particular. Whereas multiple lignin depolymerization techniques exist, the resulting bio-oils are still rich in oxygen due to the remaining alcohol, phenol, and methoxyphenyl moieties.^2,3^ These remaining functionalities increase the viscosity and lower the stability and heating values of the oils.^4^ Removing the unwanted functionalities via catalytic hydrodeoxygenation (HDO) is challenging due to the strong C–O bonds, in particular, in phenols.^5^ Selective hydrogenolysis of the Ar–O bond to form aromatic hydrocarbons is further challenged by the competing hydrogenation of the aromatic rings, resulting in H_2_ overconsumption and complete reduction to cycloalkanes.^6−8^

To overcome these problems and to help valorize lignin, numerous catalysts have been developed and tested for the HDO of lignin and lignin model compounds. Whereas transition-metal phosphides^9^ and sulfides^6^ tend to be susceptible to deactivation, carbides,^10^ oxides,^6^ and supported first-row transition metals^11^ require high temperatures and H_2_ pressures to achieve appreciable activity. Higher activity is typically obtained for oxide-supported catalysts based on Re^12−14^ and noble metals such as Ru,^15,16^ Rh,^17^ Pd,^18^ and Pt.^19^ Nevertheless, these catalysts also require high H_2_ pressures, and their selectivity for aromatic hydrocarbons is often low. Importantly, the activity and selectivity of such supported catalysts depend strongly on the synergistic effects arising from the combination of the transition-metal catalyst and the Lewis acidic support, as found, for example, for Pd@ZrO_2_,^20^ Ru@TiO_2_,^21,22^ and Ru@Nb_2_O_5_^15^ catalysts, resulting in selective Ar–OH hydrogenolysis in phenols adsorbed on the acidic support.

High selectivity for aromatics has also been achieved in the HDO of lignin-derived phenol model compounds using molecular iridium-based catalysts, although without the added benefit of recyclability.^23^ Despite the desirable selectivity that these results suggest for iridium, surprisingly few heterogeneous Ir-based HDO catalysts have been reported.^24,25^ HDO activity has been observed under a high H_2_ pressure (30 bar) for iridium supported on ZrO_2_^24^ or ZSM-5,^25^ albeit with no selectivity for aromatic compounds. Carbon also does not appear to be a suitable support for iridium in HDO, as only negligible conversion of guaiacol has been obtained.^26^ Here, we combine a proven, selectivity-enhancing support (Nb_2_O_5_)^15,27^ and a promising transition metal (Ir)^23^ that is underexplored in heterogeneously catalyzed HDO.^6,28−30^ Synergistic effects of this combination were first explored in the HDO of monoalkylated phenol compounds, which constitute the major fraction in most of the bio-oils of the so-called lignin-to-liquid (Ltl) process.^2^ Subsequently, alkylated catechol, anisole, and guaiacol, key components of bio-oils derived from lignin pyrolysis, were also tested as substrates for the Ir@Nb_2_O_5_ combination. Catalyst optimization by varying the H_2_ pressure and reaction temperature was performed using 4-cyclohexylphenol (4-CyPhOH) as a model phenol substrate for the Ltl bio-oils and to ease the analysis of the products.

## 2 Results and Discussion

The Nb_2_O_5_-supported iridium catalyst **1** (Ir@Nb_2_O_5_) was synthesized by impregnating Nb_2_O_5_ with a solution of IrCl_3_·*x*H_2_O in 40% aqueous methanol at 80 °C for 3 h, followed by reduction under H_2_ at 250 °C. A 0.62 wt % iridium loading was achieved, as determined by inductively coupled plasma-atomic emission spectroscopy (ICP-AES). Catalyst **1** exhibits type IV adsorption–desorption isotherm and H4-type hysteresis, with a specific area of 170 m^2^ g^–1^ and textural properties similar to the pristine Nb_2_O_5_ material (cf. Figures S1 and S2 in the Supporting Information). The powder X-ray diffraction pattern of catalyst **1** contains no characteristic Bragg diffraction, confirming the amorphous nature of the niobium oxide^31^ and the absence of large iridium particles (Figure S3). Corroborating this observation, the transmission electron microscopy (TEM) images of Ir@Nb_2_O_5_ reveal iridium nanoparticles with an average diameter of 1.3 ± 0.3 nm (Figure S4). Diffuse reflectance infrared Fourier transform spectroscopy (DRIFTS) of catalyst **1** (Figure S5) shows the apparition following IrCl_3_ impregnation and reduction of new O–H stretching frequencies at 3709 and 3662 cm^–1^, respectively, attributed to μ_1_-OH and μ_2_-OH groups,^32^ along with a stretching frequency band at 2337 cm^–1^ suggesting the presence of Ir–H species.^33^

First, the scope of Ir@Nb_2_O_5_ in HDO reactions was examined using a series of representative phenolic monomers (alkylated phenols, naphthol, catechol, and anisole). To our delight, in these initial tests, performed under mild conditions (*T* = 200 °C; *P*_H_2__ = 10 bar; cf. Scheme S1 for the experimental setup), mono-oxygenated phenols such as 4-cyclohexylphenol (4-CyPhOH, entry 1 in Table 1), 4-phenylphenol (4-PhPhOH, entry 2), and naphthol (entry 3) were completely converted to deoxygenated hydrocarbons in less than 10 h (see Table S1 for details), suggesting that the Ir@Nb_2_O_5_ combination indeed offers HDO-boosting synergy effects compared to both molecular Ir-based species alone^23^ and other niobia-supported metals.^15,27^

**Table 1:**
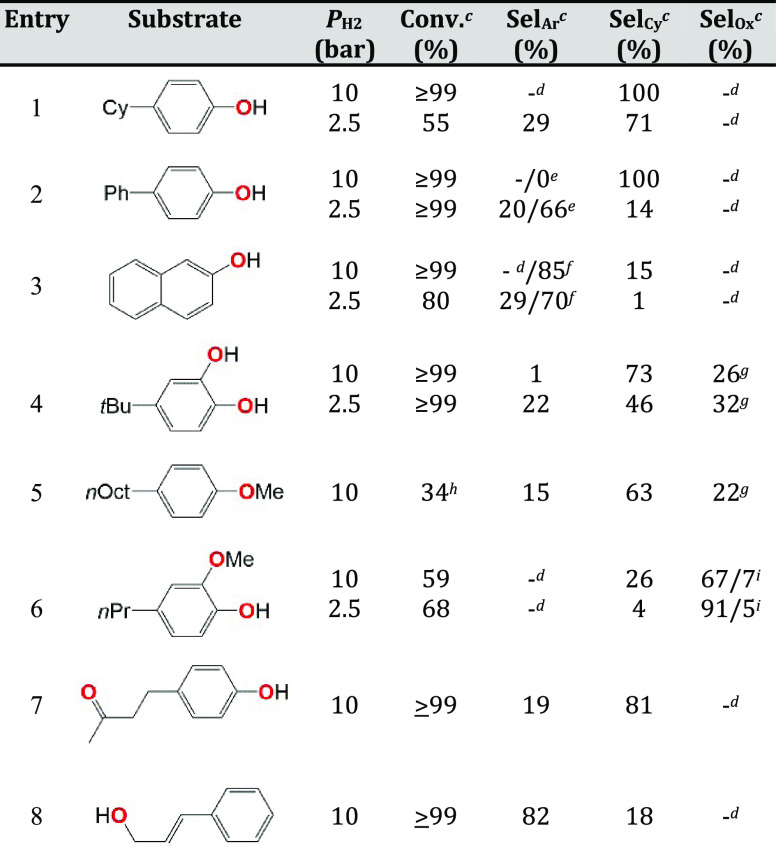
HDO of Lignin-Derived Phenols Catalyzed by Ir@Nb_2_O_5_^a^,^b^^i^

a Catalyst synthesis: impregnation of a solution of hydrated IrCl_3_ in 40% aqueous methanol onto Nb_2_O_5_ at 80 °C for 3 h, drying under vacuum, and reduction under H_2_ at 250 °C for 2 h (Ir loading: 0.62 wt %).

b Reaction conditions: 1.1 mol %_Ir_, 280 μmol of phenol derivative in 4 mL of *n*-hexadecane at 200 °C for 10 h.

c Conversion and selectivity were determined by gas chromatography-mass spectrometry (GC-MS) in THF using *n*-dodecane as the internal standard.

d Not detected.

e Phenylbenzene/cyclohexylbenzene.

f Naphthalene/tetrahydronaphthalene.

g Alkylated phenols.

h 6 h.

i 2-Methoxy-5-propylphenol/1,2-dimethoxy-4-propylbenzene.

**Scheme 1:**
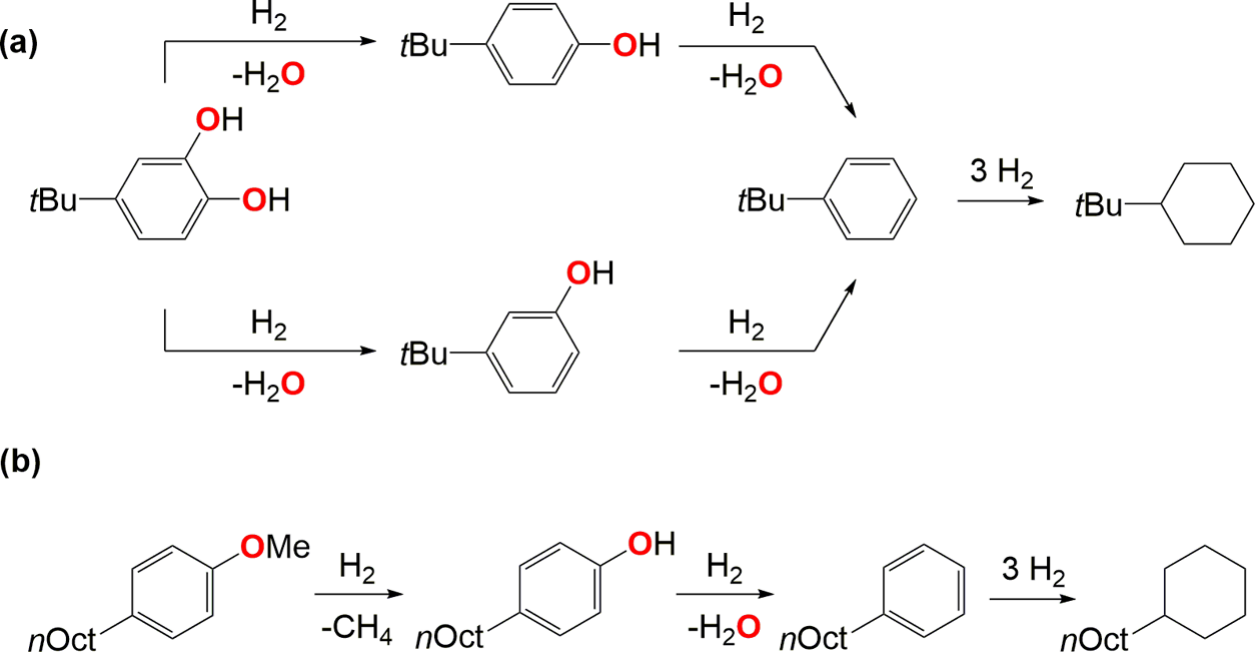
Proposed Reaction Pathways for the HDO, Demethylation, and Subsequent Hydrogenation of Alkylated (a) Catechol and (b) Anisole

Turning now to the selectivity in these early tests at *P*_H_2__ = 10 bar, fully hydrogenated products dominated (Table 1, entries 1–5), except for tetrahydronaphthalene being obtained from naphthol in an 85% yield (entry 3). The alkylated catechol and anisole substrates gave oxygenated products (Sel_Ox_ = 26 and 22% at *P*_H_2__ = 10 bar, respectively; see Table 1, entries 4 and 5) derived from either hydrogenolysis of only one Ar–OH bond or the ArO–CH_3_ bond, respectively (Scheme 1). This indicates that longer reaction times are required for a second hydrogenolysis of the remaining Ar–OH bond, as shown on the other alkylated phenols (Table 1, entries 1–3). Interestingly, unlike previous reports on Pt- and Re-based catalysts,^34−37^ no methyl transfer from the methoxy group to the aromatic ring was observed for anisole.

**Scheme 2:**
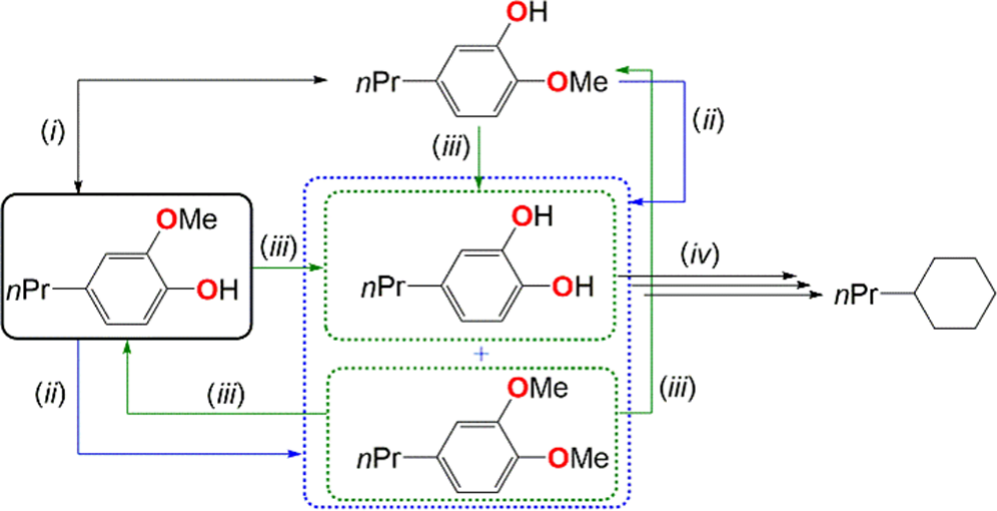
Proposed Reaction Pathway for the Isomerization and Demethylation of Guaiacol, and Subsequent HDO and Hydrogenation of Catechol (i) Intramolecular Me-transfer, (ii) intermolecular Me-transfer (+*n*Pr-guaiacol), (iii) demethylation (+H_2_, −CH_4_), and (iv) HDO and hydrogenation (+5H_2_, −2H_2_O).

For the alkylated guaiacol substrate (entry 6), a methyl transfer isomerization product, 2-methoxy-5-propylphenol, is obtained as the major product (Sel = 67%), indicating that HDO reactions leading to *n*-propyl cyclohexane (Sel_Cy_ = 26%) proceed very slowly due to a competing isomerization side reaction (Figure S6). However, after an extended reaction time (40 h), high conversion is achieved (85%; see Figure S6), and the selectivity in *n*-propyl cyclohexane reaches 64% and is concomitant to a slow decrease of 2-methoxy-5-propylphenol selectivity, confirming a slow deoxygenation process presumably involving catechol and phenol intermediates (not detected). Such isomerization reactions, observed as intramolecular Me-transfer mechanisms in enzymatic systems,^38^ seem favored over the productive HDO pathways (cleavage of ArO–CH_3_, followed by the Ar–OH cleavage via a catechol intermediate; Scheme 2). Likewise, a minor side product, 1,2-dimethoxy-4-propylbenzene, suggests that methylation of the phenol group also takes place via an intermolecular Me-transfer mechanism akin to that described for cobalamin enzyme systems,^39^ which yields a catechol intermediate (Scheme 2).

In the present case, the latter intermediate (not observed) appears to undergo rapid hydrogenation to the corresponding cycloalkane (with Sel_Cy_ = 26%). The aforementioned observations of HDO of anisole, where a small amount of phenol was formed (as shown in Table 1, entry 5), suggest that the HDO of methoxide groups (including guaiacol) proceeds via the initial rupture of the CH_3_–OAr bond (demethylation) followed by the Ar–OH cleavage, rather than a direct Ar–OCH_3_ cleavage (demethoxylation).

The relatively low yield of deoxygenated products obtained in the HDO of guaiacol suggests that the above-mentioned methyl transfer isomerization competes with, and impedes, the ArO–CH_3_ cleavage when both OH and OCH_3_ groups are present. Catalytic hydrolysis of phenols may provide a workaround for this problem.^40,41^

To further extend the reaction scope, two other substrates, i.e., 4-(4′-hydroxyphenyl)-2-butanone (raspberry ketone) and *trans*-cinnamyl alcohol, were tested (Table 1, entries 7 and 8, respectively). Both were completely converted, which demonstrates that the catalyst is able to deoxygenate substrates beyond the phenol and methoxyphenyl moieties described above. The ketone and alcohol HDO predominantly give saturated (aliphatic) and aromatic products, respectively.

**Figure 1:**
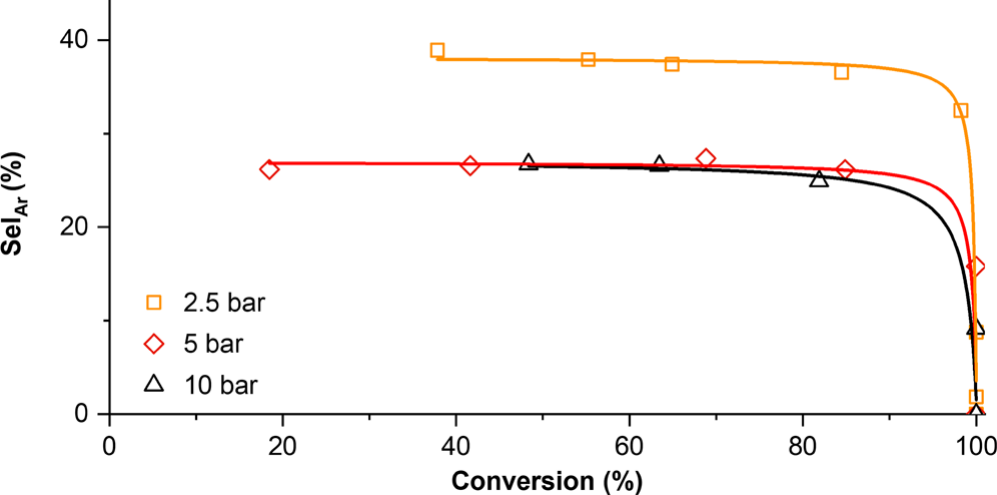
Aromatic selectivity vs conversion for 4-CyPhOH HDO at varying H_2_ pressures at 200 °C.

To further understand the influence of reaction conditions and support on the activity and the selectivity for aromatics, 4-CyPhOH was selected as a model compound, owing to its low volatility and relatively few HDO products. A blank run (200 °C, 10 bar H_2_, 10 h) using pure Nb_2_O_5_ as a catalyst yielded no measurable catalytic activity. Similarly, 0.5% Ir supported on activated carbon showed negligible activity with only trace amounts (not quantifiable by GC-MS) of bicyclohexane being produced, thus confirming the synergy between iridium nanoparticles and the Nb_2_O_5_ support. For a 10 h reaction time at 200 °C, lowering the H_2_ pressure increases the yield of cyclohexylbenzene significantly, from zero (*P*_H_2__ = 10 bar) to 29% (*P*_H_2__ = 2.5 bar) (Table 1, entry 1). The selectivity for aromatics, investigated by in situ sampling, remained essentially constant during 4-CyPhOH conversion (Figure 1), and ketones, aliphatic alcohols, or other oxygenates were not observed under any conditions.

On approaching full conversion, the reaction proceeds toward complete hydrogenation to Cy–cyclohexane (bicyclohexane), regardless of pressure. As anticipated, H_2_ pressure drastically affected kinetics, with full conversion to oxygen-free hydrocarbons being achieved in 36 and 3 h at 2.5 and 10 bar, respectively (Figure S7). In comparison, the current state-of-the-art catalyst Ru@Nb_2_O_5_ achieved an 84% conversion of 4-methylphenol in 3 h at 250 °C under 5 bar of H_2_ in aqueous solution, with cyclohexanol (10%) and cyclohexanone (2%) as side products.^15^ As for 4-CyPhOH above, reducing the H_2_ pressure to 2.5 bar drastically increases the selectivity toward aromatic products also for other alkylated phenols, catechol, and naphthol (Tables 1 and S1). Guaiacol, for which the above-described isomerization is favored at the expense of hydrogenation, is an exception.

**Figure 2:**
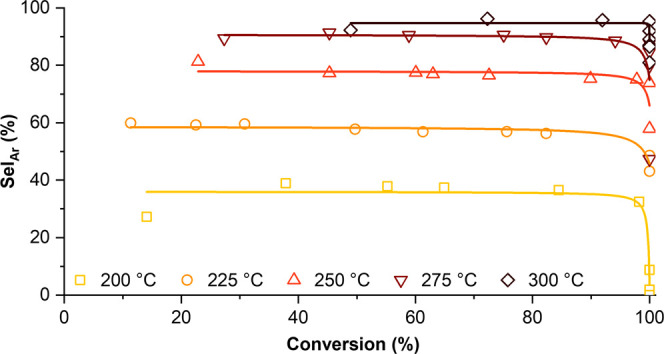
Aromatic selectivity vs conversion for 4-CyPhOH HDO at varying temperatures at 2.5 bar of H_2_.

Encouraged by the effect of reduced H_2_ pressure, we performed the HDO of 4-CyPhOH at *P*_H_2__ = 2.5 bar, with the reaction temperature being increased from 200 to 300 °C in 25 °C increments (Figure 2).

**Figure 3:**
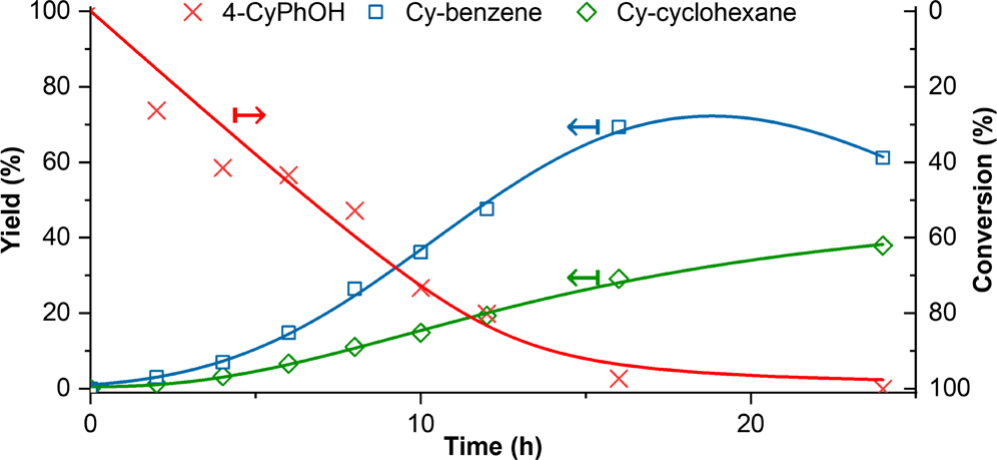
Product yields as a function of time for the HDO of 4-CyPhOH using DIPEA-treated Ir@Nb_2_O_5_ at 2.5 bar and 250 °C.

As expected, the reaction accelerates with increasing temperature, with full conversion requiring 36 h at 200 °C and only 4 h at 300 °C (Figure S8). The selectivity for aromatics is also strongly promoted by higher temperatures, reaching 95% at ≥99% conversion at 300 °C. However, above 225 °C, the product loss increases with temperature, severely limiting the overall yield of aromatics (Figure S9). This loss presumably results from coking, as previously reported for similar systems,^42^ and catalyzed by Brønsted-acidic sites,^43^ whether originally present on Nb_2_O_5_ or resulting from the residual HCl formed during IrCl_3_ reduction (cf. the Ir@Nb_2_O_5_ preparation and pH measurement described in the Supporting Information). Even though dehydroxylation is the commonly used method for the partial removal of Brønsted-acidic sites from metal oxide supports,^44^ we envisaged that such sites might be neutralized by Hünig’s base (*i*Pr_2_NEt = DIPEA).^45^ Indeed, their partial removal is confirmed by pyridine adsorption experiments (see DRIFT spectroscopy, Figures S5 and S10). TEM micrographs show a slight increase in support aggregation but no difference in the Ir particle size (Figures S4 and S11). The DIPEA-treated catalyst fully converts 4-CyPhOH at 250 °C without product loss, suggesting successful inhibition of coking, with a slightly decreased selectivity (Sel_Ar_ = 71% vs Sel_Ar_ = 77% without DIPEA treatment; Figure S12). The maximum aromatic yield is 69% for the treated catalyst, vs 37% without DIPEA treatment, and is reached after 16 h (conv. = 98%; Figure 3). Micrographs collected via TEM on the spent catalyst (Figure S11) show no significant difference in size or aggregation of either Ir or support particles.

The catalyst was recycled and used in four consecutive HDO reactions of 4-CyPhOH at 225 °C and 2.5 bar of H_2_ without significant reduction in selectivity (Figure S13). Reduced catalytic activity, ascribed to the loss of material during the recycling process, was observed after the second catalyst recovery.

## 3 Conclusions

In conclusion, we describe the first easy-to-prepare and recyclable heterogeneous, Nb_2_O_5_-supported iridium HDO catalyst. The catalyst efficiently and selectively converts naphthol, and alkylated phenols, catechols, anisoles, and, to a lesser extent, guaiacols, to hydrocarbons under mild conditions (down to 2.5 bar of H_2_ and 200 °C). The new iridium-based catalyst compares well to state-of-the-art systems such as Nb_2_O_5_-supported ruthenium,^15^ especially in terms of selectivity for hydrocarbons, as no oxygenated products were obtained in the HDO of *mono*-phenols. Although the selectivity for aromatics from such substrates is relatively low at moderate H_2_ pressure (e.g., 25%), lower pressure and higher reaction temperature improve the selectivity toward aromatics (up to 95%), albeit at the cost of significant product loss (up to 80%), presumably resulting from coking. Using Hünig’s base, coking and product loss could be prevented by a novel method for the selective removal of Brønsted-acidic sites, allowing for high aromatic yields (up to 69%) under these otherwise challenging conditions. Further work will focus on the structure–activity and structure–selectivity relationships, the reaction mechanisms, and screening and optimization of the oxide support, followed by the upgrading of phenol-enriched bio-oils from Ltl processes.

## 4 Experimental Section

### Preparation of Ir@Nb_2_O_5_

The Ir@Nb_2_O_5_ catalyst was synthesized by IrCl_3_ impregnation followed by reduction under H_2_ at 250 °C. Hydrated Nb_2_O_5_ (7.53 g) was added to a solution of IrCl_3_·*x*H_2_O (1.33 mmol, with *x* ≈ 6) in 40% aqueous methanol. The suspension was briefly sonicated and stirred at 80 °C for 4 h. The solids were then filtered, washed with methanol (3 × 20 mL), and vacuum-dried. Reduction was performed by heating the material thus obtained at 250 °C under 5 bar (absolute) of H_2_ for 2 h, followed by vacuum at 250 °C for another 2 h. Elemental analysis found (wt %): Ir: 0.62.

Measurement of pH was performed by stirring and sonicating Ir@Nb_2_O_5_ (0.25 g) in distilled water (10 mL). The resulting suspension was found to be acidic with a pH of 5.1. Filtration through a syringe filter (0.45 μm PTFE membrane) leads to a clear solution. Addition of AgNO_3_ (0.14 g in 0.2 mL H_2_O) to 1.3 mL of that solution resulted in very light opalescence, suggesting the precipitation of AgCl.

### Pyridine Adsorption on Ir@Nb_2_O_5_

In a glovebox under an inert atmosphere, the Ir@Nb_2_O_5_ catalyst sample was placed in a pear-shaped flask, which was then connected to a T-shaped bridge and a Schlenk flask containing 10 mL of pyridine (dried over calcium hydride, degassed by freeze–pump–thaw). The assembled glassware was then taken outside of the glovebox, connected to a Schlenk line, and the catalyst flask was immersed in an oil bath at 150 °C and evacuated. Under a static vacuum, the pyridine flask was opened, allowing the vapors to fill the system and adsorb on the catalyst. After 1 h of adsorption, the pyridine flask was closed, and the catalyst was put under a dynamic vacuum for 2 h, to remove any excess or weakly adsorbed pyridine. The sample was then handled and stored under an inert atmosphere in a glovebox.

### Preparation of *i*Pr_2_NEt (Hünig’s Base) onto Ir@Nb_2_O_5_

Selective removal of Brønsted-acidic sites with *i*Pr_2_NEt (DIPEA) was performed under argon. Reduced Ir@Nb_2_O_5_ (1 g) and dried DIPEA (2 mL) were added to a pear-shaped flask and stirred for 16 h. The sample was then washed with moisture-free dichloromethane (6 × 10 mL) and dried under vacuum.

### Typical Procedure for HDO Reaction

Each catalytic run was performed by adding Ir@Nb_2_O_5_ (100 mg) and 4-CyPhOH (0.28 mmol) to a glass pressure tube (1.1 mol %_Ir_), alongside 4 mL of *n*-hexadecane as a solvent and *n*-dodecane (12 mg) as an internal reference. The contents were stirred and sonicated, degassed under vacuum, and flushed under the desired H_2_ pressure. The reaction was started by increasing a preheated aluminum heating block around the glass tube. To minimize product losses through evaporation, the upper part of the glass tube was equipped with a metal insert, and the top of the reactor was heated up to 170 °C. The in situ sampling was performed at desired intervals by dipping a stainless steel tube in the reaction mixture through a septum and opening a valve, allowing the H_2_ pressure to push some of the solution through the tube and into a collection flask; then, the sampling tube was flushed with H_2_. A detailed schematic of the reactor setup used is provided in Scheme S1.

### Recycling Procedure for HDO Reaction

Catalyst recyclability tests were performed using Ir@Nb_2_O_5_ (100 mg) and 4 mL of a stock solution containing 12.5 g L^–1^ of 4-CyPhOH and 3 g L^–1^ of dodecane in *n*-hexadecane. Due to the limited solubility of 4-CyPhOH in *n*-hexadecane, the stock solution was preheated to 100 °C and stirred before each use to ensure homogeneity. Catalyst and stock solution were added to the glass pressure tube, and the reaction was conducted at 225 °C under 2.5 bar H_2_ (absolute) for a duration of 3 h. No in situ sampling was performed. Once the reaction was completed, the contents of the reactor were allowed to decant at 100 °C, a small amount of the supernatant was sampled, the catalyst and supernatant were then transferred to a centrifuge tube, separated by centrifugation, and then the catalyst was washed twice with hot hexadecane (100 °C). The washed catalyst was then placed back into the pressure tube, and the process was repeated. Results are given in Figure S7.

## Supporting Information Available

Experimental and analytical details, DRIFT spectra, PXRD diffractogram, TEM image, and catalytic tests (PDF)

## Abbreviations

HDO: hydrodeoxygenation
Ltl: lignin-to-liquid
4-PhPhOH: 4-phenylphenol
4-CyPhOH: 4-cyclohexylphenol
Cy–cyclohexane: bicyclohexane
Cy–benzene: cyclohexylbenzene
DIPEA: diisopropylamine

## References

[ref1] Bajwa D. S.; Pourhashem G.; Ullah A. H.; Bajwa S. G. A Concise Review of Current Lignin Production, Applications, Products and Their Environmental Impact. Ind. Crops Prod. 2019, 139, 111526 10.1016/j.indcrop.2019.111526.

[ref2] Kleinert M.; Barth T. Phenols from Lignin. Chem. Eng. Technol. 2008, 31, 736–745. 10.1002/ceat.200800073.

[ref3] Pires A. P. P.; Arauzo J.; Fonts I.; Domine M. E.; Arroyo A. F.; Garcia-Perez M. E.; Montoya J.; Chejne F.; Pfromm P.; Garcia-Perez M. Challenges and Opportunities for Bio-Oil Refining: A Review. Energy Fuels 2019, 33, 4683–4720. 10.1021/acs.energyfuels.9b00039.

[ref4] Lu Q.; Li W.-Z.; Zhu X.-F. Overview of Fuel Properties of Biomass Fast Pyrolysis Oils. Energy Convers. Manage. 2009, 50, 1376–1383. 10.1016/j.enconman.2009.01.001.

[ref5] Furimsky E. Catalytic Hydrodeoxygenation. Appl. Catal., A 2000, 199, 147–190. 10.1016/S0926-860X(99)00555-4.

[ref6] Zhang J.; Sun J.; Wang Y. Recent Advances in the Selective Catalytic Hydrodeoxygenation of Lignin-Derived Oxygenates to Arenes. Green Chem. 2020, 22, 1072–1098. 10.1039/C9GC02762A.

[ref7] Zhao C.; Lercher J. A. Selective Hydrodeoxygenation of Lignin-Derived Phenolic Monomers and Dimers to Cycloalkanes on Pd/C and HZSM-5 Catalysts. ChemCatChem 2012, 4, 64–68. 10.1002/cctc.201100273.

[ref8] Kong J.; He M.; Lercher J. A.; Zhao C. Direct Production of Naphthenes and Paraffins from Lignin. Chem. Commun. 2015, 51, 17580–17583. 10.1039/C5CC06828B. 26478925

[ref9] Ivars-Barceló F.; Asedegbega-Nieto E.; Aguado E. R.; Cecilia J. A.; Molina A. I.; Rodríguez-Castellón E. 6. Advances in the Application of Transition Metal Phosphide Catalysts for Hydrodeoxygenation Reactions of Bio-Oil from Biomass Pyrolysis. In Biomass and Biowaste; De Gruyter, 2020; pp 145–166.

[ref10] Tran C. C.; Han Y.; Garcia-Perez M.; Kaliaguine S. Synergistic Effect of Mo-W Carbides on Selective Hydrodeoxygenation of Guaiacol to Oxygen-Free Aromatic Hydrocarbons. Catal. Sci. Technol. 2019, 9, 1387–1397. 10.1039/C8CY02184H.

[ref11] Huynh T. M.; Armbruster U.; Pohl M. M.; Schneider M.; Radnik J.; Hoang D. L.; Phan B. M. Q.; Nguyen D. A.; Martin A. Hydrodeoxygenation of Phenol as a Model Compound for Bio-Oil on Non-Noble Bimetallic Nickel-Based Catalysts. ChemCatChem 2014, 6, 1940–1951. 10.1002/cctc.201402011.

[ref12] Ghampson I. T.; Pecchi G.; Fierro J. L. G.; Videla A.; Escalona N. Catalytic Hydrodeoxygenation of Anisole over Re-MoO_x_/TiO_2_ and Re-VO_x_/TiO_2_ Catalysts. Appl. Catal., B 2017, 208, 60–74. 10.1016/j.apcatb.2017.02.047.

[ref13] Leiva K.; Martinez N.; Sepulveda C.; García R.; Jiménez C. A.; Laurenti D.; Vrinat M.; Geantet C.; Fierro J. L. G.; Ghampson I. T.; Escalona N. Hydrodeoxygenation of 2-Methoxyphenol over Different Re Active Phases Supported on SiO2 Catalysts. Appl. Catal., A 2015, 490, 71–79. 10.1016/j.apcata.2014.10.054.

[ref14] Herrera C.; Ghampson I. T.; Cruces K.; Sepúlveda C.; Barrientos L.; Laurenti D.; Geantet C.; Serpell R.; Contreras D.; Melin V.; Escalona N. Valorization of Biomass Derivatives through the Conversion of Phenol over Silica-Supported Mo-Re Oxide Catalysts. Fuel 2020, 259, 116245 10.1016/j.fuel.2019.116245.

[ref15] Shao Y.; Xia Q.; Dong L.; Liu X.; Han X.; Parker S. F.; Cheng Y.; Daemen L. L.; Ramirez-Cuesta A. J.; Yang S.; Wang Y. Selective Production of Arenes via Direct Lignin Upgrading over a Niobium-Based Catalyst. Nat. Commun. 2017, 8, 16104 10.1038/ncomms16104. 28737172 PMC5527281

[ref16] Yao G.; Wu G.; Dai W.; Guan N.; Li L. Hydrodeoxygenation of Lignin-Derived Phenolic Compounds over Bi-Functional Ru/H-Beta under Mild Conditions. Fuel 2015, 150, 175–183. 10.1016/j.fuel.2015.02.035.

[ref17] Resende K. A.; Noronha F. B.; Hori C. E. Hydrodeoxygenation of Phenol over Metal Supported Niobia Catalysts. Renewable Energy 2020, 149, 198–207. 10.1016/j.renene.2019.12.061.

[ref18] de Souza P. M.; Rabelo-Neto R. C.; Borges L. E. P.; Jacobs G.; Davis B. H.; Resasco D. E.; Noronha F. B. Hydrodeoxygenation of Phenol over Pd Catalysts. Effect of Support on Reaction Mechanism and Catalyst Deactivation. ACS Catal. 2017, 7, 2058–2073. 10.1021/acscatal.6b02022.

[ref19] Lee H.; Kim H.; Yu M. J.; Ko C. H.; Jeon J.-K.; Jae J.; Park S. H.; Jung S.-C.; Park Y.-K. Catalytic Hydrodeoxygenation of Bio-Oil Model Compounds over Pt/HY Catalyst. Sci. Rep. 2016, 6, 28765 10.1038/srep28765. 27357731 PMC4928091

[ref20] de Souza P. M.; Rabelo-Neto R. C.; Borges L. E. P.; Jacobs G.; Davis B. H.; Graham U. M.; Resasco D. E.; Noronha F. B. Effect of Zirconia Morphology on Hydrodeoxygenation of Phenol over Pd/ZrO_2_. ACS Catal. 2015, 5, 7385–7398. 10.1021/acscatal.5b01501.

[ref21] Duan H.; Liu J.-C.; Xu M.; Zhao Y.; Ma X.-L.; Dong J.; Zheng X.; Zheng J.; Allen C. S.; Danaie M.; Peng Y.-K.; Issariyakul T.; Chen D.; Kirkland A. I.; Buffet J.-C.; Li J.; Tsang S. C. E.; O’Hare D. Molecular Nitrogen Promotes Catalytic Hydrodeoxygenation. Nat. Catal. 2019, 2, 1078–1087. 10.1038/s41929-019-0368-6.

[ref22] Nelson R. C.; Baek B.; Ruiz P.; Goundie B.; Brooks A.; Wheeler M. C.; Frederick B. G.; Grabow L. C.; Austin R. N. Experimental and Theoretical Insights into the Hydrogen-Efficient Direct Hydrodeoxygenation Mechanism of Phenol over Ru/TiO_2_. ACS Catal. 2015, 5, 6509–6523. 10.1021/acscatal.5b01554.

[ref23] Kusumoto S.; Nozaki K. Direct and Selective Hydrogenolysis of Arenols and Aryl Methyl Ethers. Nat. Commun. 2015, 6, 6296 10.1038/ncomms7296. 25704229

[ref24] Alda-Onggar M.; Mäki-Arvela P.; Aho A.; Simakova I. L.; Murzin D. Y. Hydrodeoxygenation of Phenolic Model Compounds over Zirconia Supported Ir and Ni-Catalysts. React. Kinet., Mech. Catal. 2019, 126, 737–759. 10.1007/s11144-018-1502-1.

[ref25] Pawelec B.; Loricera C. V.; Geantet C.; Mota N.; Fierro J. L. G.; Navarro R. M. Factors Influencing Selectivity in the Liquid-Phase Phenol Hydrodeoxygenation over ZSM-5 Supported Pt/Ir and Pt+Ir Catalysts. Mol. Catal. 2020, 482, 110669 10.1016/j.mcat.2019.110669.

[ref26] Chang J.; Danuthai T.; Dewiyanti S.; Wang C.; Borgna A. Hydrodeoxygenation of Guaiacol over Carbon-Supported Metal Catalysts. ChemCatChem 2013, 5, 3041–3049. 10.1002/cctc.201300096.

[ref27] Shao Y.; Xia Q.; Liu X.; Lu G.; Wang Y. Pd/Nb_2_O_5_/SiO_2_ Catalyst for the Direct Hydrodeoxygenation of Biomass-Related Compounds to Liquid Alkanes under Mild Conditions. ChemSusChem 2015, 8, 1761–1767. 10.1002/cssc.201500053. 25876904

[ref28] Bu Q.; Lei H.; Zacher A. H.; Wang L.; Ren S.; Liang J.; Wei Y.; Liu Y.; Tang J.; Zhang Q.; Ruan R. A Review of Catalytic Hydrodeoxygenation of Lignin-Derived Phenols from Biomass Pyrolysis. Bioresour. Technol. 2012, 124, 470–477. 10.1016/j.biortech.2012.08.089. 23021958

[ref29] Jin W.; Pastor-Pérez L.; Shen D.; Sepúlveda-Escribano A.; Gu S.; Ramirez Reina T. Catalytic Upgrading of Biomass Model Compounds: Novel Approaches and Lessons Learnt from Traditional Hydrodeoxygenation - a Review. ChemCatChem 2019, 11, 924–960. 10.1002/cctc.201801722.

[ref30] Qu L.; Jiang X.; Zhang Z.; Zhang X.; Song G.; Wang H.; Yuan Y.; Chang Y. A Review of Hydrodeoxygenation of Bio-Oil: Model Compounds, Catalysts, and Equipment. Green Chem. 2021, 23, 9348–9376. 10.1039/D1GC03183J.

[ref31] da Conceição L. R. V.; Carneiro L. M.; Rivaldi J. D.; de Castro H. F. Solid Acid as Catalyst for Biodiesel Production via Simultaneous Esterification and Transesterification of Macaw Palm Oil. Ind. Crops Prod. 2016, 89, 416–424. 10.1016/j.indcrop.2016.05.044.

[ref32] Jeantelot G.; Ould-Chikh S.; Sofack-Kreutzer J.; Abou-Hamad E.; Anjum D. H.; Lopatin S.; Harb M.; Cavallo L.; Basset J.-M. Morphology Control of Anatase TiO_2_ for Well-Defined Surface Chemistry. Phys. Chem. Chem. Phys. 2018, 20, 14362–14373. 10.1039/C8CP01983E. 29767182

[ref33] Martinez-Macias C.; Chen M.; Dixon D. A.; Gates B. C. Single-Site Zeolite-Anchored Organoiridium Carbonyl Complexes: Characterization of Structure and Reactivity by Spectroscopy and Computational Chemistry. Chem. - Eur. J. 2015, 21, 11825–11835. 10.1002/chem.201501277. 26140330

[ref34] Saidi M.; Rostami P.; Rahimpour H. R.; Roshanfekr Fallah M. A.; Rahimpour M. R.; Gates B. C.; Raeissi S. Kinetics of Upgrading of Anisole with Hydrogen Catalyzed by Platinum Supported on Alumina. Energy Fuels 2015, 29, 4990–4997. 10.1021/acs.energyfuels.5b00297.

[ref35] Sudhakar P.; Pandurangan A. Pt/Ni Wet Impregnated over Al Incorporated Mesoporous Silicates: A Highly Efficient Catalyst for Anisole Hydrodeoxygenation. J. Porous Mater. 2018, 25, 747–759. 10.1007/s10934-017-0488-9.

[ref36] Ghampson I. T.; Canales R.; Escalona N. A Study of the Hydrodeoxygenation of Anisole over Re-MoO_x_/TiO_2_ Catalyst. Appl. Catal., A 2018, 549, 225–236. 10.1016/j.apcata.2017.10.009.

[ref37] Lai Q.; Zhang C.; Holles J. H. Mo@Pt Overlayers as Efficient Catalysts for Hydrodeoxygenation of Guaiacol and Anisole. Catal. Sci. Technol. 2017, 7, 3220–3233. 10.1039/C7CY00565B.

[ref38] Farnberger J. E.; Hiebler K.; Bierbaumer S.; Skibar W.; Zepeck F.; Kroutil W. Cobalamin-Dependent Apparent Intramolecular Methyl Transfer for Biocatalytic Constitutional Isomerization of Catechol Monomethyl Ethers. ACS Catal. 2019, 9, 3900–3905. 10.1021/acscatal.8b05072. 31080689 PMC6503581

[ref39] Farnberger J. E.; Richter N.; Hiebler K.; Bierbaumer S.; Pickl M.; Skibar W.; Zepeck F.; Kroutil W. Biocatalytic Methylation and Demethylation via a Shuttle Catalysis Concept Involving Corrinoid Proteins. Commun. Chem. 2018, 1, 82 10.1038/s42004-018-0083-2.

[ref40] Podschun J.; Saake B.; Lehnen R. Catalytic Demethylation of Organosolv Lignin in Aqueous Medium Using Indium Triflate under Microwave Irradiation. React. Funct. Polym. 2017, 119, 82–86. 10.1016/j.reactfunctpolym.2017.08.007.

[ref41] Yang L.; Li Y.; Savage P. E. Hydrolytic Cleavage of C–O Linkages in Lignin Model Compounds Catalyzed by Water-Tolerant Lewis Acids. Ind. Eng. Chem. Res. 2014, 53, 2633–2639. 10.1021/ie403545n.

[ref42] Shafaghat H.; Rezaei P. S.; Daud W. M. A. W. Catalytic Hydrodeoxygenation of Simulated Phenolic Bio-Oil to Cycloalkanes and Aromatic Hydrocarbons over Bifunctional Metal/Acid Catalysts of Ni/HBeta, Fe/HBeta and NiFe/HBeta. J. Ind. Eng. Chem. 2016, 35, 268–276. 10.1016/j.jiec.2016.01.001.

[ref43] Zanuttini M. S.; Dalla Costa B. O.; Querini C. A.; Peralta M. A. Hydrodeoxygenation of M-Cresol with Pt Supported over Mild Acid Materials. Appl. Catal., A 2014, 482, 352–361. 10.1016/j.apcata.2014.06.015.

[ref44] Le Roux E.; Anwander R. Surface Organolanthanide and -Actinide Chemistry. In Modern Surface Organometallic Chemistry; Wiley-VCH Verlag GmbH & Co. KGaA: Weinheim, Germany, 2009; Vol. 1, pp 455–512.

[ref45] Hünig S.; Kiessel M. Spezifische Protonenacceptoren Als Hilfsbasen Bei Alkylierungs- Und Dehydrohalogenierungsreaktionen. Chem. Ber. 1958, 91, 380–392. 10.1002/cber.19580910223.

