# Supplementary material for: Selective Hydrodeoxygenation of Lignin-Derived Phenols to Aromatics Catalyzed by Nb2O5-Supported Iridium
Source: ACS Omega. 2022 Aug 23;7(35):31561–6. doi: 10.1021/acsomega.2c04314 (PMC9453801; doi:10.1021/acsomega.2c04314)
Supplement: Supplementary file 1 — ao2c04314_si_001.pdf [file ao2c04314_si_001.pdf]

Supporting information

Selective Hydrodeoxygenation of Lignin-Derived Phenols to Aromatics Catalyzed by Nb_2O_5-Supported Iridium

Gabriel Jeantelot,* Simen P. Følkner, Johanna I. S. Manegold, Morten G. Ingebrigtsen, Vidar R. Jensen, Erwan Le Roux*

General procedure

4-Cyclohexyl-phenol (4-CyPhOH) was purchased from Ambinter. Hydrated Nb_2O_5 was obtained from Companhia Brasileira de Metalurgia e Mineração (CBMM, referenced as HY-340) with a BET surface area of 170 m^2 g^{-1} after applying a thermo-vacuum treatment at 250 °C for 4 h. 0.5% Ir/C was purchased from Fisher Scientific, and vacuum-dried prior use. All other chemicals and solvents were purchased from Sigma-Aldrich and used as received without further purification. Hydrogen was purchased from Yara Praxair (purity grade: 99.999%).

X-ray diffractograms were acquired using a Bruker D8-advance diffractometer, from 2θ = 4° to 2θ = 90°, 2500 steps, 0.5 s integration per step.

TEM images were acquired on a Hitachi HT7800 microscope with a 100 kV acceleration voltage and 2.2 µA emission current.

Gas adsorption measurements were carried out on a BELSORP-max instrument. The sample was then dried in a dynamic vacuum at 200 °C overnight.

FT-IR spectra were recorded with a Nicolet 460 spectrometer equipped with a SpectraTech diffuse reflectance infrared Fourier transform (DRIFT) spectroscopy accessory with a deuterated triglycine sulfate detector. Calibration was performed with KBr. The sample was prepared in an airtight DRIFT cell with KBr windows. Spectra were recorded at room temperature with a scanning speed of 0.1581 cm^{-1} min^{-1}, an aperture of 100 and a spectral resolution of 1.928 cm^{-1}. Data was processed using the OMNIC software.

Elemental analysis of iridium content was provided from Microanalytisches Labor Pascher, and obtained via Inductively Coupled Plasma – Atomic Emission Spectrometry (ICP-AES).

Gas chromatography – Mass Spectrometry (GC-MS) was carried out using an Agilent 7890A gas chromatograph equipped with an Agilent 19091S-433 column (fused silica capillary, ID = 0.25 mm, stationary phase 5%-phenyl-methylpolysiloxane, film thickness = 0.25 µm) connected to an Agilent 5977A mass spectrometry detector. The injection volume was 0.5 µL at a carrier gas flow of 0.8 mL min^{-1} helium (4.8 psi) with a splitless injection. The initial oven temperature of 40 °C was maintained for 2.5 min and then raised to 100 °C at 20 °C min^{-1}. Then it was increased at 5 °C min^{-1} to 150 °C and kept stable or 10 more min. The temperature was held for 1 min and was then raised to 320 °C at 30 °C min^{-1} for 5 min. Other settings were a 320 °C interface temperature, 230 °C ion source temperature and electron impact ionization (EI) at 70 eV and 300 µA. Mass spectra were analyzed in the range of 20-450 atomic mass units (amu) at a rate of 3.4 scans sec^{-1} for a total run time of 37.167 min (including 5 min solvent delay).

Synthesis of 4-*n*-octylanisole [CAS: 3307-19-5]

4-*n*-octylanisole was synthetized according to a different procedure compared to the literature procedure.[1-4] 4-*n*-Octylphenol (232.3 mg), K_2CO_3 (235.5 mg) and dimethylformamide (1 mL) were added to a glass pressure tube, and cooled to 0 °C in an ice bath. Under stirring, methyl iodide (71 µL) was added dropwise. The reagents were then stirred overnight. Deionized water (4 mL) was then added under vigorous stirring and extracted with *n*-hexane (4 mL). The organic phase was washed with a saturated aqueous solution of NaCl (2 × 4 mL), filtered through a 0.45 µm syringe filter (PVDF, hydrophobic), and dried under vacuum overnight, resulting in a colorless oil (95% yield estimated by ^1H NMR). ^1H NMR (500.13 MHz, $CDCl_3$): δ 7.10 (m, 2H, Ar-*H*), 6.84 (2H, Ar-*H*), 3.80 (s, 3H, O-CH_3), 2.55 (m, 2H, Ar-CH_2-CH_2-$(CH_2)_5$-CH_3), 1.58 (m, 2H, Ar-CH_2-CH_2-$(CH_2)_5$-CH_3), 1.29 (m, 10H, Ar-CH_2-CH_2-(CH_2)$_5$-CH_3), 0.89 (t, 3H, Ar-CH_2-CH_2-$(CH_2)_5$-CH_3) ppm. The ^1H NMR data matched those reported in the literature.[1-4]

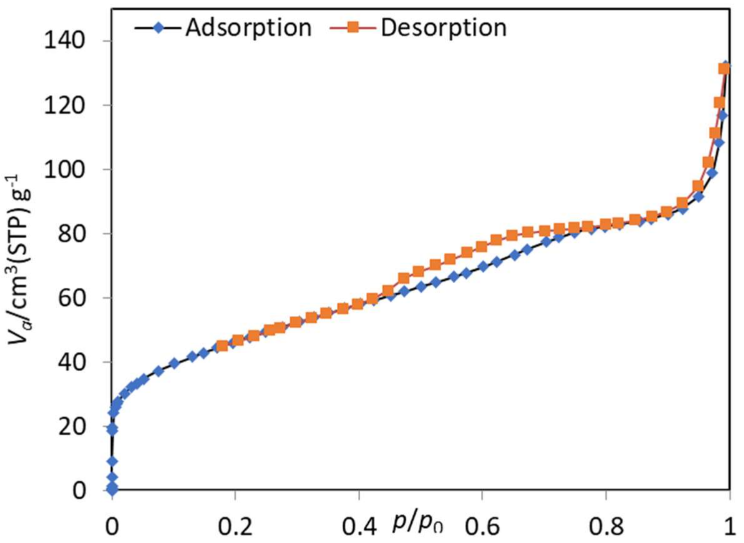

Figure S1. N_2 adsorption isotherms of the Nb_2O_5 support.

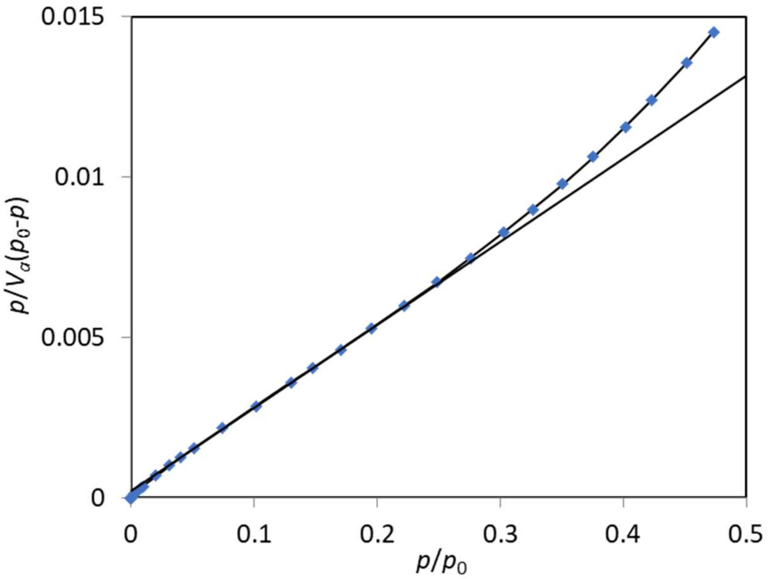

Figure S2. BET plot of N_2 adsorption on the Nb_2O_5 support.

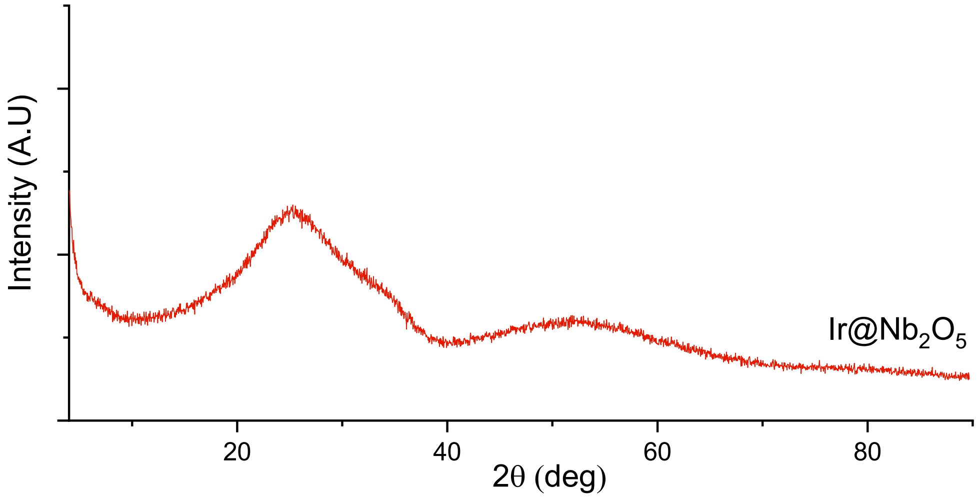

Figure S3. Powder X-ray diffractogram of $Ir@Nb_2O_5$.

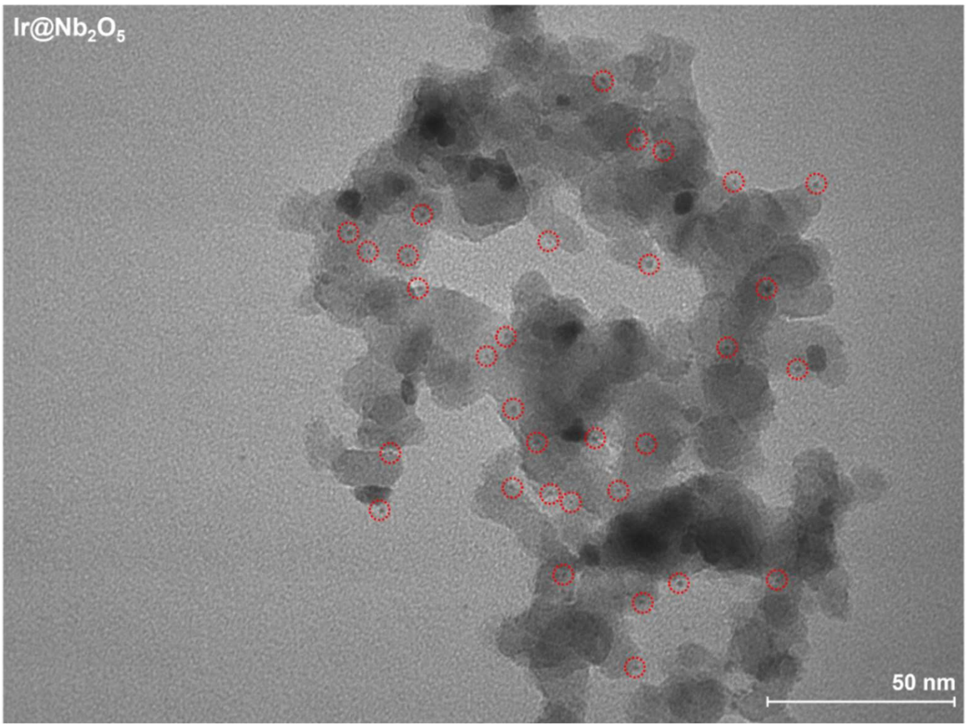

Figure S4. TEM micrographs of $Ir@Nb_2O_5$. Red circles denote Ir nanoparticles.

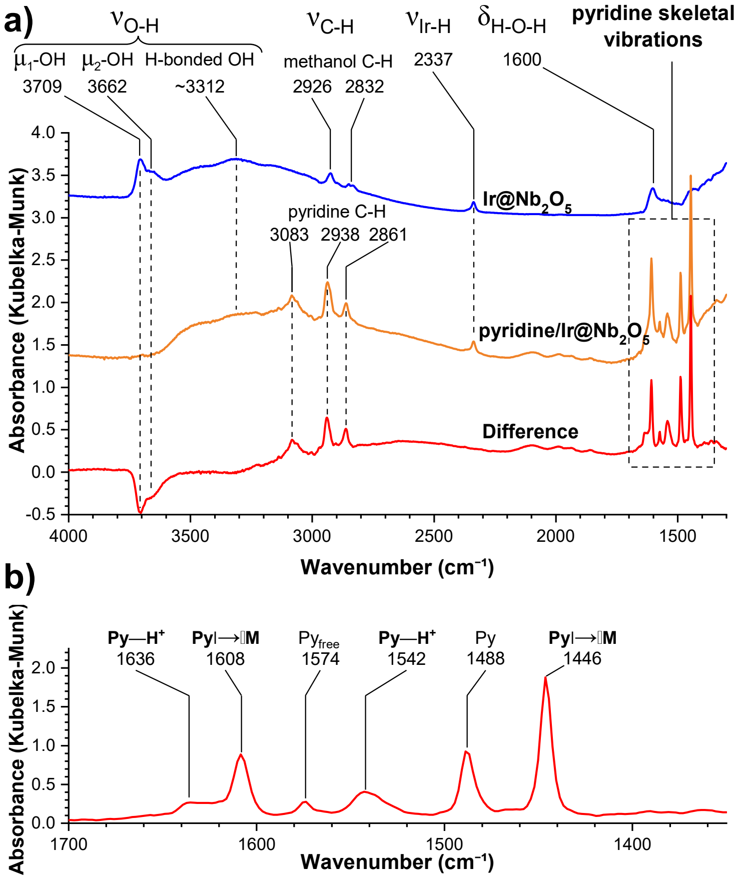

Figure S5. a) DRIFT spectra of Ir@Nb$_2$O$_5$ before and after pyridine adsorption ($\nu_{Ir\text{-}H}$ = 2337 cm^{-1}).[5] b) Detail and attribution of the pyridine adsorption peaks. Remark: Pyridine adsorption at 150 °C leads to IR bands characteristic of pyridine coordinated to on Lewis-acidic sites (1608 cm^{-1}, 1446 cm^{-1}) and Brønsted-acidic sites (1636 cm^{-1}, 1542 cm^{-1}).[6] The absorbance of the 3709 and 3662 cm^{-1} O-H stretching bands is strongly reduced by pyridine adsorption, suggesting they constitute the support's Brønsted acidic sites.

Scheme S1. Reactor setup for catalysis & in-situ sampling

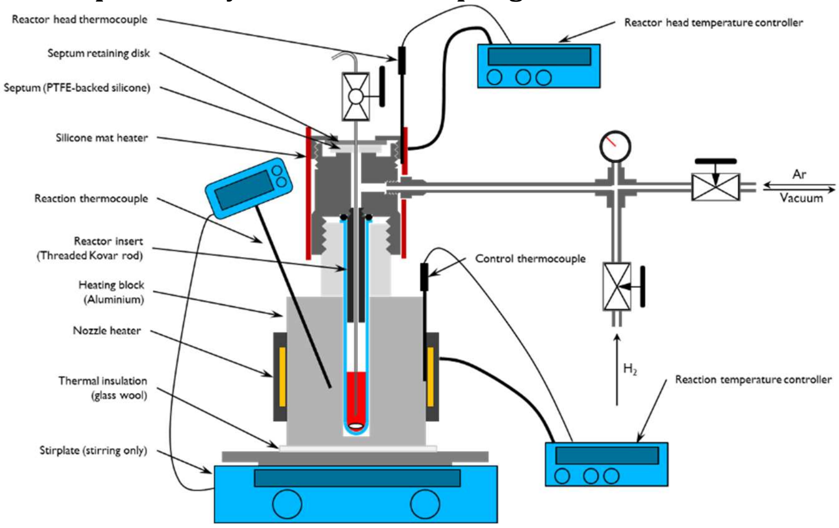

Table S1. HDO of lignin-derived phenols catalyzed by Ir@Nb_2O_5.[a,b]

Substrate	Time (h)	P_{H2} (bar)	Conv.[c] (%)	Sel$_{Ar}$[c] (%) Full. aromatic	Sel$_{Ar}$[c] (%) Part. aromatic	Sel$_{Cy}$[c] (%)	Sel$_{Ox}$[c] (%)
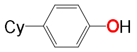	10	10	≥99	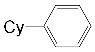 0		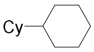 100	
		2.5	41	38		62	
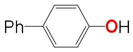	10	10	≥99	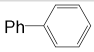 0	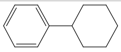 0	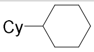 100	
		2.5	≥99	20	66	14	
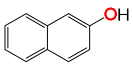	10	10	≥99	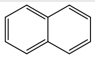 0	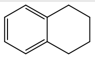 85	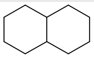 15	
		2.5	80	29	70	1	
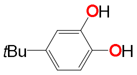	10	10	≥99	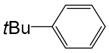 1		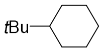 73	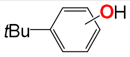 26
		2.5	≥99	22		46	33
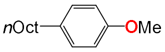	6	10	34	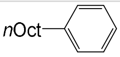 15		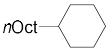 63	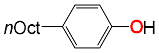 22
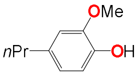	10	10	59	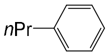 0		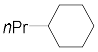 26	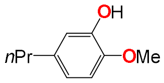 67 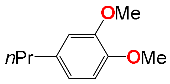 7
		2.5	68	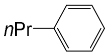 0		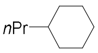 4	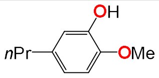 91 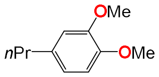 5
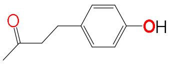	10	10	≥99	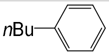 19		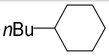 81	
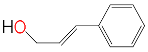	10	10	≥99	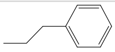 82		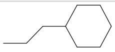 18	

[a] Catalyst formation: impregnation of a solution of hydrated $IrCl_3$ in 40% aqueous methanol onto Nb_2O_5 at 80 °C for 3 h, dried under vacuum, and reduced under H_2 at 250 °C for 2 h (Ir loading: 0.62 wt%). [b] Reaction conditions: 1.1 mol%$_{Ir}$, 280 μmol of derived-phenols in 4 mL *n*-hexadecane at 200 °C. [c] Conversion and selectivity were determined by GC-MS in THF using *n*-dodecane as internal standard.

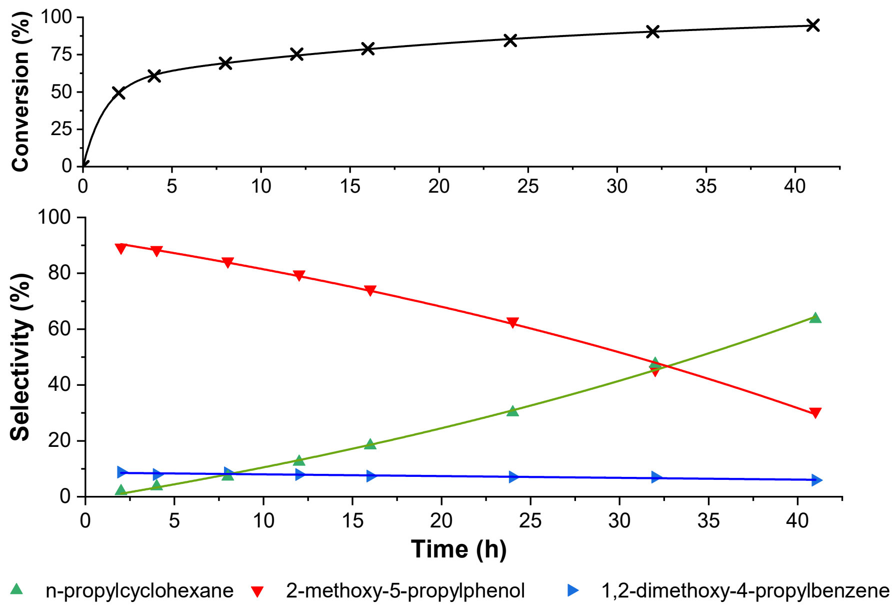

Figure S6. Kinetic study of 4-propylguaiacol hydrodeoxygenation.

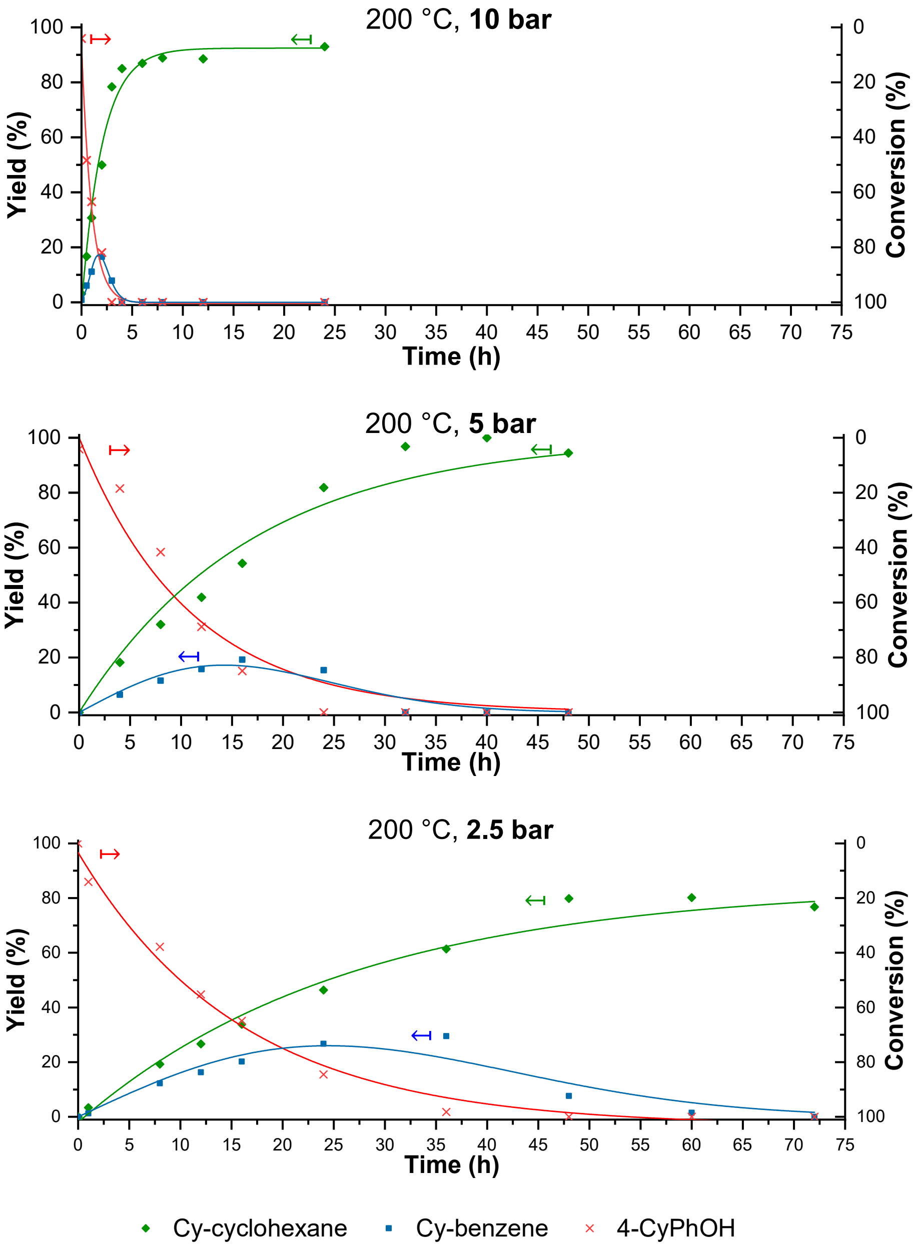

Figure S7. HDO of 4-CyPhOH: products yield as a function of time at varying H_2 pressures.

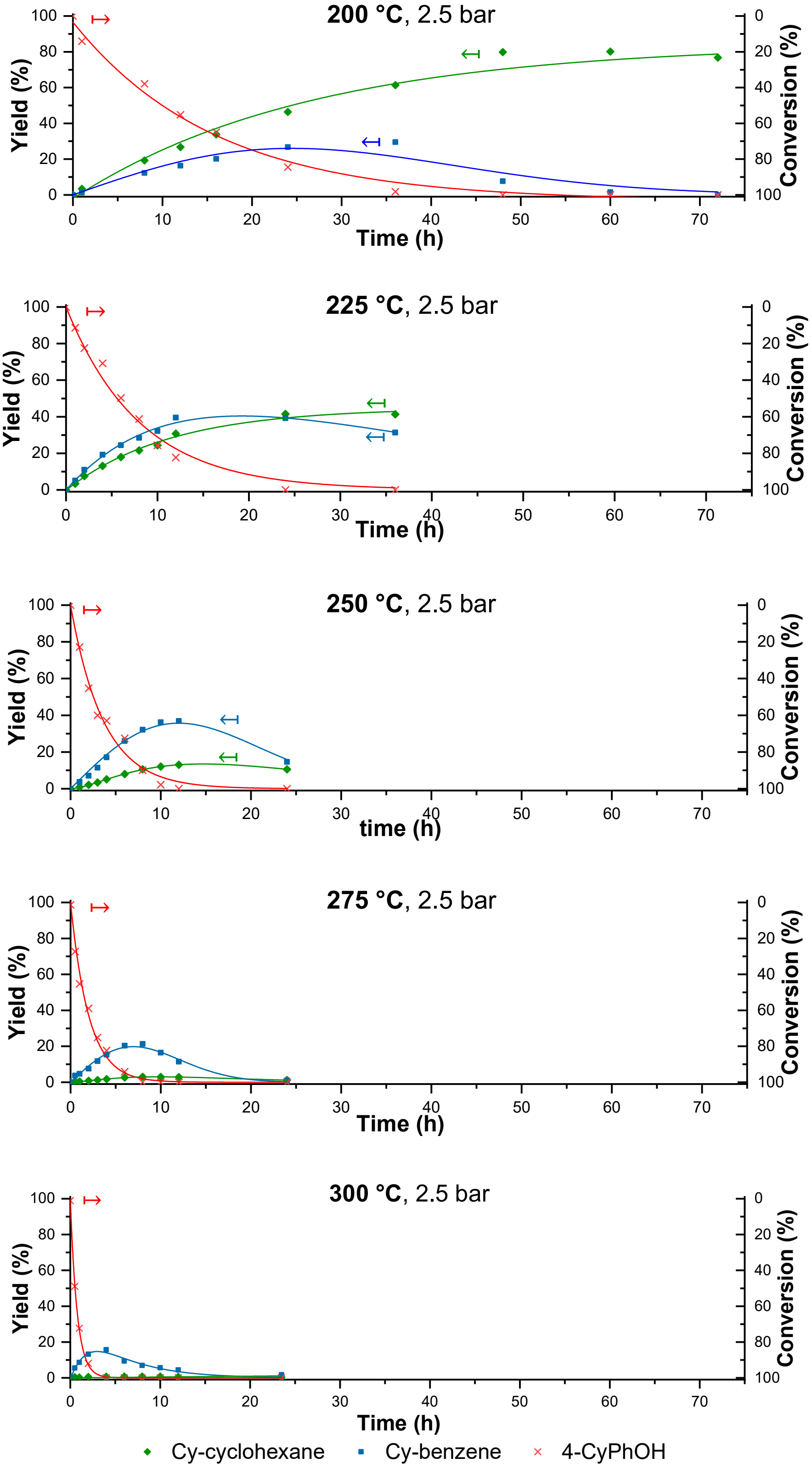

Figure S8. HDO of 4-CyPhOH: products yield as a function of time at varying reaction temperatures.

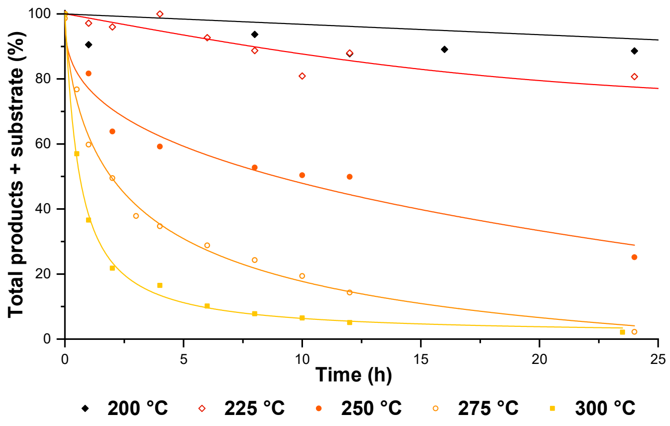

Figure S9. HDO of 4-CyPhOH: overall product loss over time at 2.5 bar H_2.

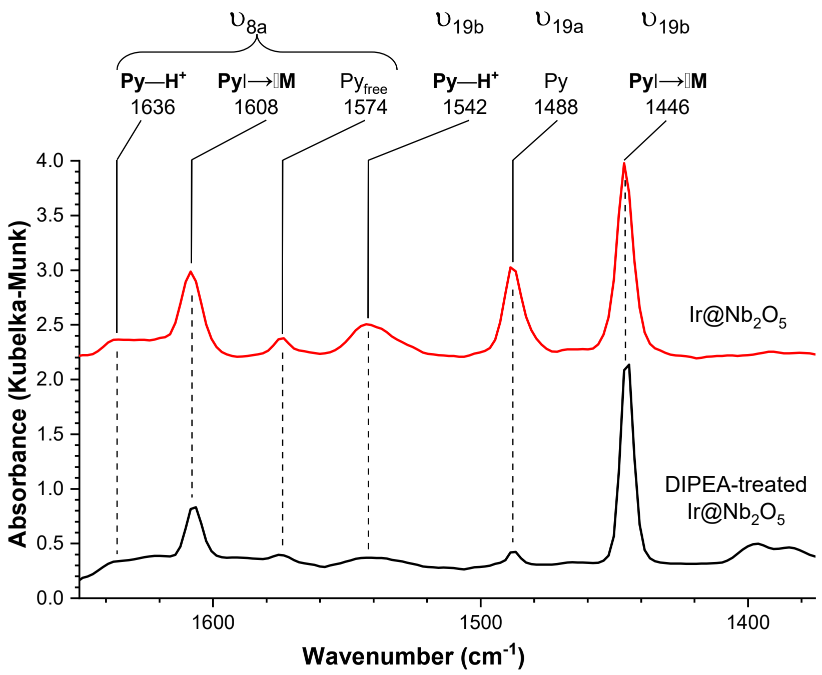

Figure S10. DRIFT spectra of pyridine adsorbed on $Ir@Nb_2O_5$ with and without DIPEA treatment.

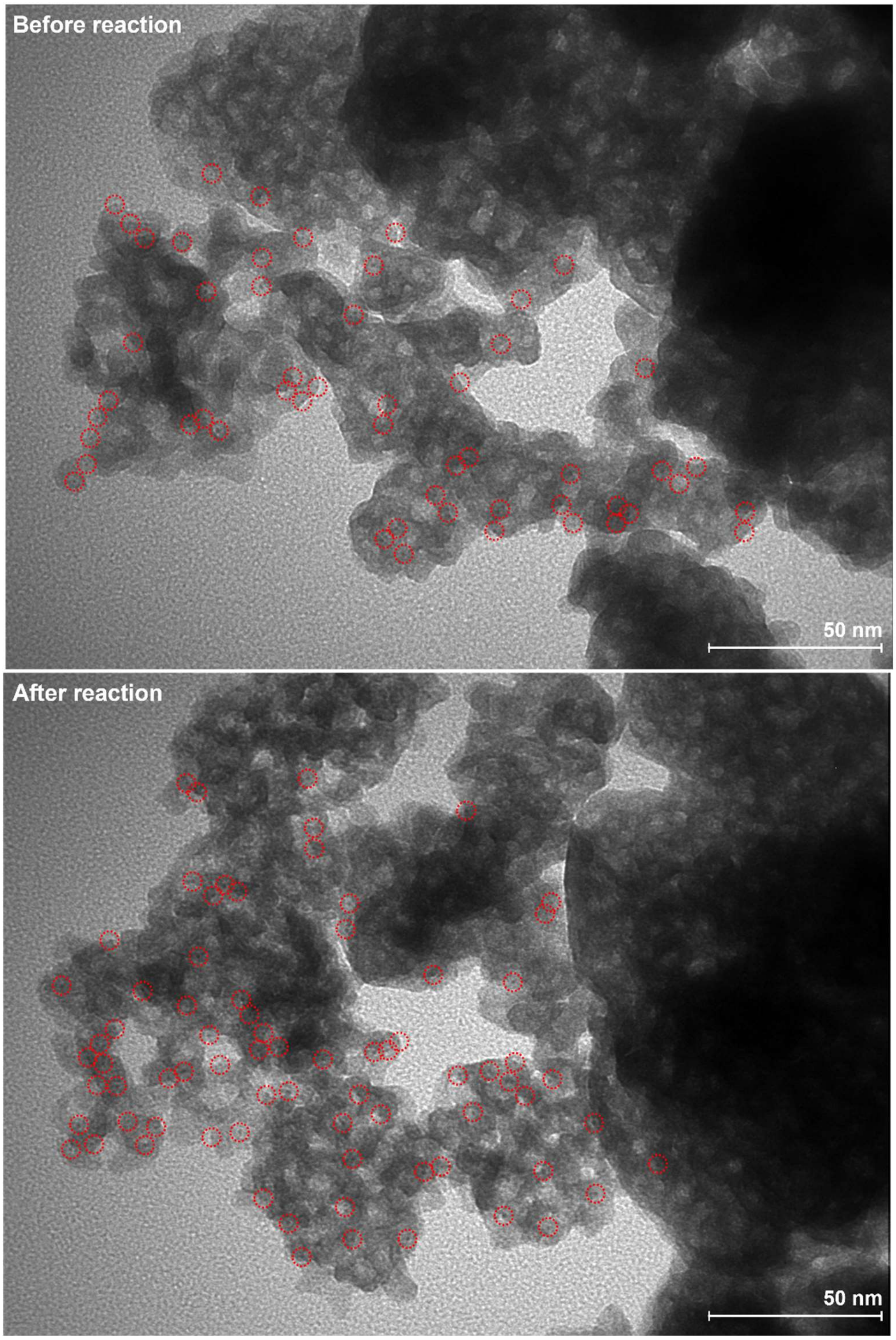

Figure S11. TEM micrographs of DIPEA-treated Ir@Nb_2O_5 before (top) and after HDO reaction (bottom). Reaction conditions: DIPEA-treated Ir@Nb_2O_5 (100 mg), 4-CyPhOH (0.28 mmol), 4 mL n-hexadecane, H_2 (2.5 bar), 16 h at 250 °C, with *n*-dodecane as internal standard.

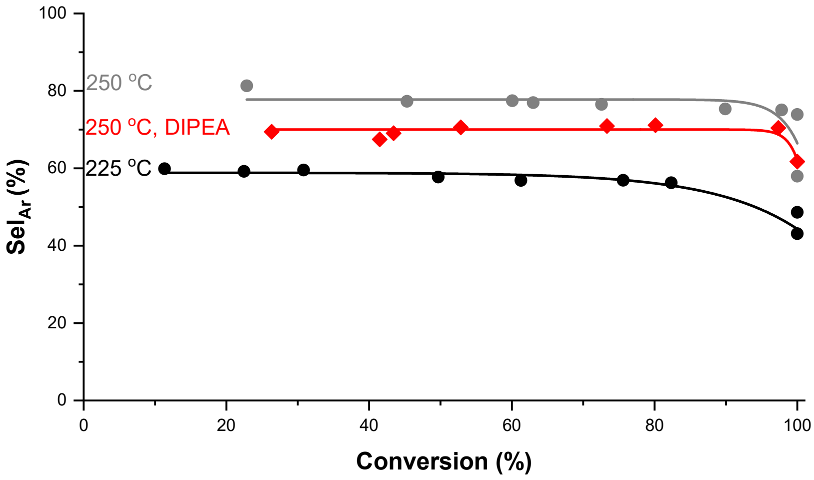

Figure S12. HDO of 4-CyPhOH: selectivity in aromatics vs conversion curves with DIPEA-treated Ir@Nb_2O_5 (250 °C, 2.5 bar H_2) compared with Ir@Nb_2O_5 without DIPEA treatment (225-250°C, 2.5 bar H_2).

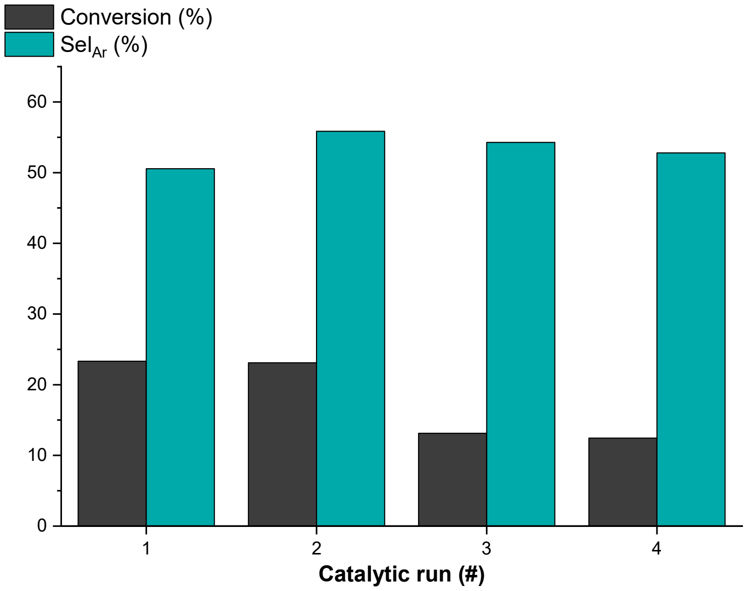

Figure S13. Catalyst recyclability test results.

References:

1. Dreher, S. D.; Lim, S.-E.; Sandrock, D. L.; Molander, G. A., Suzuki−Miyaura Cross-Coupling Reactions of Primary Alkyltrifluoroborates with Aryl Chlorides. *J. Org. Chem.* **2009,** *74* (10), 3626-3631.
2. Cahiez, G.; Chaboche, C.; Duplais, C.; Moyeux, A., A New Efficient Catalytic System for the Chemoselective Cobalt-Catalyzed Cross-Coupling of Aryl Grignard Reagents with Primary and Secondary Alkyl Bromides. *Org. Lett.* **2009,** *11* (2), 277-280.
3. Dai, W.-M.; Li, Y.; Zhang, Y.; Yue, C.; Wu, J., Generation of an Aromatic Amide-Derived Phosphane (Aphos) Library by Self-Assisted Molecular Editing and Applications of Aphos in Room-Temperature Suzuki–Miyaura Reactions. *Chem. Eur. J.* **2008,** *14* (18), 5538-5554.

4. Everson, D. A.; Shrestha, R.; Weix, D. J., Nickel-Catalyzed Reductive Cross-Coupling of Aryl Halides with Alkyl Halides. *J. Am. Chem. Soc.* **2010,** *132* (3), 920-921.
5. Martinez-Macias, C.; Xu, P.; Hwang, S.-J.; Lu, J.; Chen, C.-Y.; Browning, N. D.; Gates, B. C., Iridium Complexes and Clusters in Dealuminated Zeolite HY: Distribution between Crystalline and Impurity Amorphous Regions. *ACS Catal.* **2014,** *4* (8), 2662-2666.
6. Layman, K. A.; Ivey, M. M.; Hemminger, J. C., Pyridine Adsorption and Acid/Base Complex Formation on Ultrathin Films of γ-Al2O3 on NiAl(100). *J. Phys. Chem. B* **2003,** *107* (33), 8538-8546.
